# Vacancy Ordering in Fe-Deficient Iron Sulfide with the NiAs-Type Structure

**DOI:** 10.1021/acs.jpcc.4c05199

**Published:** 2025-04-04

**Authors:** David Santos-Carballal, Nora H. de Leeuw

**Affiliations:** † School of Chemistry, University of Leeds, Leeds LS2 9JT, United Kingdom; ‡ Department of Earth Sciences, Utrecht University, 3584 CD Utrecht, The Netherlands

## Abstract

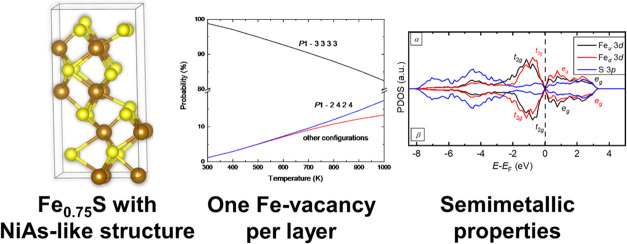

An Fe-deficient iron sulfide thin film with a nickeline (NiAs) type structure has been reported with a stoichiometry close to greigite (Fe_3_S_4_) [DavisE. M.; Phys. Chem. Chem. Phys.2019, 21, 20204–2021031486466 10.1039/c9cp04157e]. We have investigated the Fe-vacancy ordering in the nonstoichiometric iron sulfide with the NiAs-like structure using density functional theory calculations with a Hubbard Hamiltonian and long-range dispersion corrections [DFT + *U* – D3(BJ)]. We applied canonical statistical mechanics to study the thermodynamics of ordering and in the most stable configuration we found the same concentration of Fe deficiencies in each layer along the *c* axis. We discuss the probabilities of the configurations and the averages of observables, such as lattice parameters and magnetic moments, as a function of temperature. At equilibrium, the Fe-deficient iron sulfide is expected to be fully ordered. The predicted electronic properties of the most stable configuration suggest that this material is antiferromagnetic. The simulated electronic structure shows that the most stable configuration of the Fe-deficient iron sulfide has semimetallic properties.

## 1 Introduction

Since the first report of the crystal structure of the nickeline (NiAs) group mineral in 1923,^1^ it has also been observed in a wide range of compositions for the first-series of transition metal chalcogenides, including achávalite (FeSe),^2^ breithauptite (NiSb),^2^ freboldite (CoSe),^3^ and others.^4,5^ Binary NiAs, which has only been found to form stoichiometric crystals,^6,7^ is the aristotype of myriad ionic compounds of reduced symmetry, such as the first-row transition metal chalcogenides FeS,^8^ NiS,^9^ VS,^10^ V_3_S_4_,^11^ V_5_S_8_,^11^ CrSe,^12^ Fe_3_Se_4_,^13^ V_3_Se_4_,^14^ and Cr_2_Te_3_,^15^ which display a range of cation/anion ratios between 1:1 and 2:3. The nonstoichiometric Cr_2.8_Se_4_,^16^ Fe_1–*x*_S (*x* = 0.06, 0.015, and 0.025),^17,18^ δ-Fe_0.96_Se,^19^ V_0.87_S,^20^ V_2.724_S_4_,^11^ TiSe_1.05_,^21^ and TiSe_0.95_,^21^ which are hettotypes of the high-symmetry NiAs structure, are of particular scientific interest owing to the presence of cation or anion vacancies.

**Figure 1:**
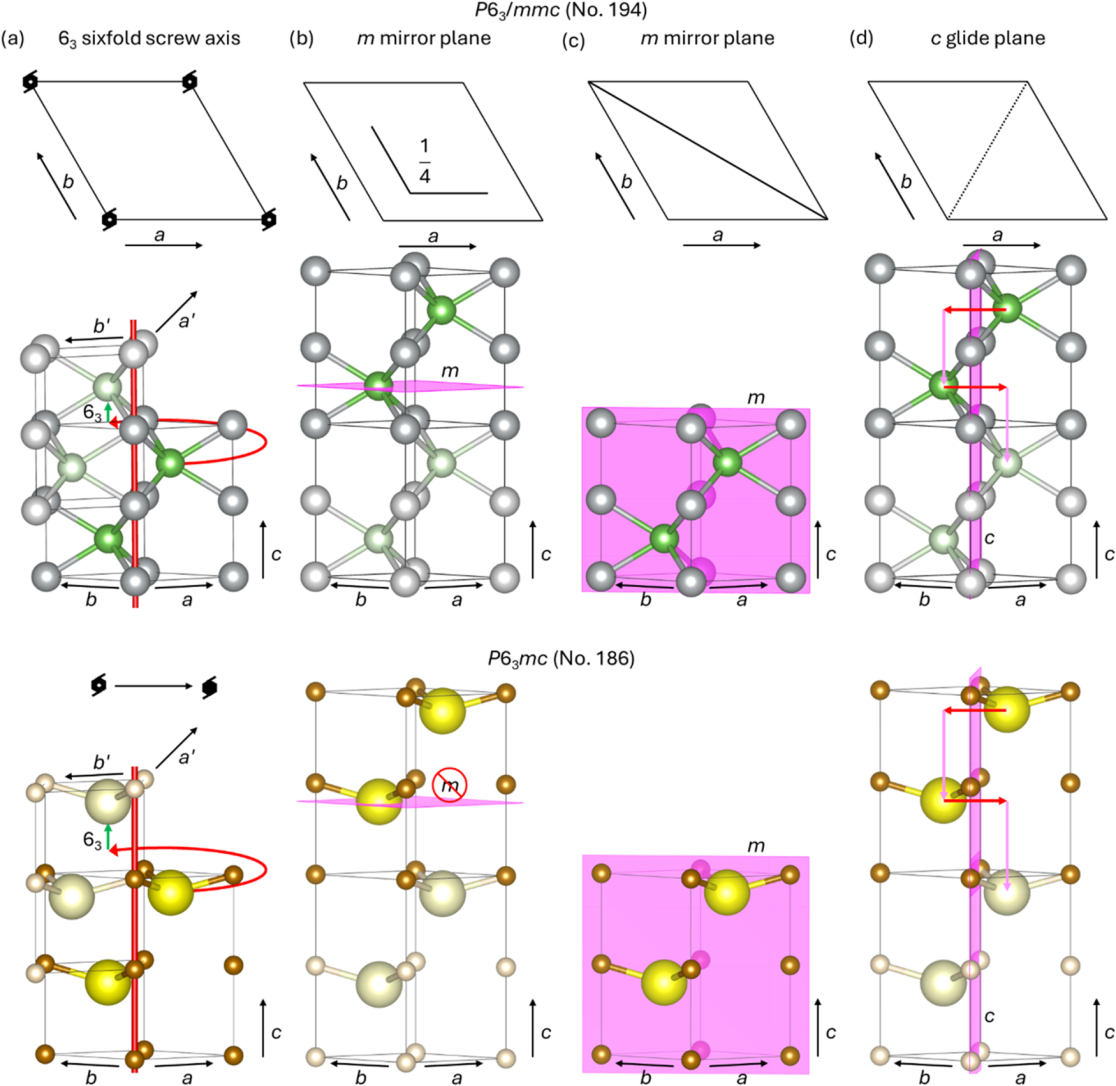
Symmetry elements explicitly present in (top panels) NiAs and (bottom panels) FeS with space groups *P*6_3_/*mmc* (No. 194) and *P*6_3_*mc* (No. 186), respectively, (a) 6_3_ sixfold screw axis, (b), and (c) *m* mirror planes, and (d) *c* glide plane. The S anions were shifted to the coordinate (1/3, 2/3, 0.4) to aid the visualisation of the missing mirror plane. Ni atoms are in grey, As atoms are in green, Fe atoms are in brown and S atoms are in yellow. The atoms of the duplicated cells are shown in lighter colour. Crystallographic directions are indicated.

NiAs, which crystallises in the last and most symmetric space group *P*6_3_/*mmc* (No. 194) of the hexagonal crystal systems,^1^ comprises two embedded sublattices. The centrosymmetric NiAs structure, which has a dihexagonal dipyramidal point group (Schön. *D*_6*h*_), contains the main sixfold screw axis 6_3_ parallel to *c*, two mirror planes *m*, one at the height of 1/4 above the *ab* plane, and other parallel to *c*, as well as a glide plane with a translation of 1/2 along *c*, as the only symmetry operations explicitly present in the space group, see top panels in Figure 1. The As anions, which have the formal −3 oxidation state and are occupying the 2*c* Wyckoff positions with coordinates (1/3, 2/3, 1/4), form a hexagonal close-packed (hcp) sublattice. The Ni cations, which have the formal +3 oxidation state and are located at the 2*a* Wyckoff position with coordinates (0, 0, 0), form a primitive hexagonal (hP) sublattice. Both Ni and As atoms have a sixfold coordination environment, forming distorted octahedra and triangular prisms, respectively. The conventional hexagonal unit cell of NiAs contains two formula units (f.u.) and the stacking sequence of the atomic layers is *AcBcAcBc*, where the *A* and *B* sites of the hcp lattice are occupied by the As atoms and the eclipsed *c* sites are occupied by Ni. There is also an alternative “anti-NiAs” configuration, where the cations instead occupy the 2*c* Wyckoff positions of the As atoms and the anions fill the 2*a* Wyckoff sites of the Ni atoms, which has been reported at least for NbN_0.95_.^22,23^

Of particular significance is the recently reported structure of Fe-deficient iron sulfide thin films by Freund and co-workers.^24^ The Fe occupancy was estimated at 74 ± 9% following X-ray diffraction (XRD) analysis or between 82 and 86% using X-ray photoelectron spectroscopy (XPS),^24^ making the stoichiometry of the thin film very close to the naturally occurring greigite (Fe_3_S_4_) mineral^25^ or smythite (Fe_9_S_11_ or Fe_13_S_16_),^26−28^ respectively. The authors of reference^24^ are cautious in the interpretation of the larger Fe occupancies obtained from XPS and hypothesise (*i*) that a significant error margin in the quantification of the peak area may be responsible, or (*ii*) that the concentration of the defects changes within the 25 nm depth of the thin film, with the film/substrate interface being Fe-poor and the exposed surface being Fe-rich. The iron sulfide thin film, which has an NiAs-type structure with Fe vacancies, belongs to the space group *P*6_3_*mc* (No. 186) and the polar dihexagonal pyramidal point group (Schön. *C*_6*v*_), differing from its parent material in the absence of the mirror plane *m* at the height of 1/4 above the *ab* plan, see bottom panels in Figure 1. The Fe cations lie in the same positions occupied by the Ni atoms in the NiAs structure, whereas the S anions are displaced to the 2*b* Wyckoff positions with coordinates (1/3, 2/3, 0.26). Note that the bottom panels in Figure 1 display the FeS structure, where we have exaggerated the displacement of the S anions to the coordinate (1/3, 2/3, 0.4) to aid the visualisation of the missing mirror plane. The Fe-deficient Fe_0.74_S, which was grown on a gold Au(111) substrate surface and was characterised using an array of surface science techniques, did not indicate the ordering of the cation vacancies. Moreover, NiAs is also the aristotype of several polytypes of iron sulfides, including pyrrhotite (Fe_1–*x*_S, 0 ≤ *x* ≤ 0.125),^18,26,29−31^ troilite (FeS),^32,33^ smythite (Fe_9_S_11_ or Fe_13_S_16_),^26−28^ and the high-temperature forms of hexagonal FeS. Both the inverse spinel greigite,^25^ which is the *quasi*-polymorph of Fe_0.74_S,^24^ and pyrite (FeS_2_)^34^ form cubic crystals, whereas their metastable precursor mackinawite (Fe_1+*x*_S, 0 ≤ *x* ≤ 0.11) crystallises in the tetragonal lattice system.^35^ Despite also forming orthorhombic crystals, marcasite (FeS_2_) does not display a NiAs-like structure,^36^ as its disulfide S_2_^2–^ groups form a different arrangement around the Fe cations than in its polymorph pyrite (FeS_2_).

The cubane-structured (Fe_4_S_4_) catalytic centre of greigite has been found to be capable of converting CO_2_ towards small quantities of organic molecules such as formic acid, acetic acid, methanol and pyruvic acid,^37^ which has lent support to several hypotheses for the origin of life.^38,39^ Moreover, mackinawite can dissociate spontaneously, from a thermodynamic point of view, the CO_2_ molecule into CO and O fragments.^40^ Freund and collaborators have reported that the as-grown Fe-deficient iron sulfide thin films with the NiAs-like structure, which are terminated with a layer of S atoms, are unable to activate the CO_2_ molecule due to the lack of exposed Fe ions.^41^ Attempts to increase the reactivity towards CO_2_ using atomic H to reduce the surface of the iron sulfide thin films were unsuccessful, as the S atoms at the topmost layer were only partially removed.^41^ However, increasing the concentration of the cation by adding metallic Fe over the surface of the iron sulfide with the NiAs-like structure was found to improve the interaction with the CO_2_ molecule.^41^

The disorder of Fe vacancies in the nonstoichiometric iron sulfide compounds is a complex phenomenon that is not yet well understood. In this paper, we present a first-principles investigation of the thermodynamics of vacancy ordering in the Fe-poor Fe_0.74_S that is stable in the inner section of the thin film, where we have simulated the geometries and stabilities, as well as the electronic and magnetic properties for different configurations of the 25% Fe vacancies in FeS with the NiAs-type structure. Modelling the distribution of Fe vacancies in Fe_0.74_S is important to understand its surface reconstructions and the chemical reactivity.

## 2 Computational Methods

The Vienna Ab Initio Simulation Package (VASP)^42−45^ was employed to simulate the effect of epitaxial strain on the vacancy ordering in the Fe-deficient Fe_0.75_S with the NiAs-type structure. We have used the generalised gradient approximation (GGA) for the exchange-correlation energy functional proposed by Perdew, Burke, and Ernzerhof (PBE) for all our spin-polarised calculations based on the density functional theory (DFT).^46,47^ Projected augmented-wave (PAW) pseudopotentials,^48,49^ including the nonspherical contributions of the density gradient within the one-centre terms, were used to model the frozen core electrons of Fe and S, *i.e.*, the [Ne]3*s*^2^3*p*^6^ and [He]2*s*^2^2*p*^4^ levels, respectively, and their interaction with the valence states. We used a modified Pulay method^50^ for the charge density mixing with the inverse Kerker metric and the Kerker preconditioner,^51^ which included up to the *g* orbitals of the one-centre PAW charge densities. The linear mixing coefficients used in the Kerker function to calculate the charge and magnetisation densities were fixed at 0.40 and 1.60, respectively. The cutoff wave vectors for the Kerker mixing scheme were determined automatically for both charge and magnetisation densities in such a way that the weight of the shortest wave vector is 20 times stronger than the longest wave vector. We allowed a minimal mixing parameter of 0.10 for the Kerker preconditioning and a maximum of 45 vectors stored in the Pulay mixer, which was reset after each ionic step. We used the Kosugi algorithm,^52^ which is a special case of the blocked Liu–Davidson iteration scheme,^53−55^ for the relaxation of the electronic degrees of freedom until the energy difference in two consecutive self-consistent (SC) loop steps dropped below 10^–8^ eV. A kinetic energy cutoff of 520 eV was applied for the periodic plane-wave basis set used to expand the Kohn–Sham (KS) valence states. The D3 semiempirical method of Grimme with Becke–Johnson (BJ) damping was included in our calculations to correct the long-range dispersion interactions and improve the description of nonbonded distances and noncovalent interaction energies.^56,57^ A simplified rotationally invariant GGA Hubbard parameter,^58,59^*U*_eff_ = 1.00 eV, was employed to enhance the simulation of the electron correlations in the 3*d* levels of the Fe cations.^60^ The DFT + *U* method, while valuable for correcting self-interaction errors in standard density functional theory, is not without its limitations. *U*_eff_ values are typically determined by fitting to experimental observables such as band gaps, magnetic moments, or heats of oxidation, or derived using the linear response approach. These values can range from 0 eV, commonly used for pure metals, up to several eV for transition metal atoms in semiconducting or insulating oxides, or in sulfides such as those studied here. However, due to the lack of relevant experimental data for the iron sulfide with the NiAs-like structure investigated in this work, we considered that it would be difficult to fit a specific *U*_eff_ value for this material and have therefore employed a *U*_eff_ value from the literature that was developed for a quasi-polymorph iron sulfide material. We consider that although not perfect, our approach captures the trends of the vacancy disorder in the iron sulfide with the NiAs-like structure under investigation, which is usually the main deliverable from theoretical materials chemistry and crystallography studies.

The initial electronic configuration was calculated through a random initialisation of the trial wave functions for an initial charge density fixed to the superposition of atomic pseudo charge densities. We kept the initial KS Hamiltonian fixed during the first five electronic SC steps to obtain reasonable orbitals and we performed a minimum of two electronic SC steps during the diagonalisation of the KS Hamiltonian in subsequent ionic steps. The nonlocal part of the PAW projection operators was evaluated in the reciprocal space to obtain accurate energies. We carried out a simple extrapolation of the charge density using their atomic components every time that the ionic configuration was changed. An efficient and memory conserving symmetrisation of the charge density was used, and we calculated the Harris corrections to the stress tensor and forces. We used the Methfessel–Paxton scheme^61^ order one with a sigma value σ = 0.11 eV to determine the electronic partial occupancies during all our geometry optimisations. The Rayleigh–Ritz scheme was used to diagonalise the Hamiltonian matrix in the subspace spanned by the wave functions. Unoccupied bands, which were defined as those with an electronic population smaller than 1 × 10^–3^ e, were optimised twice, whereas occupied bands were optimised up to four times until the change in eigen-energy was smaller than 1.4 × 10^–11^ eV or if the energy change was 30% smaller than the energy change in the first iterative optimisation step.

We used a conjugate gradient algorithm to perform the geometry optimisations,^62,63^ where we allowed the relaxation of the internal positions, cell shape and cell volume. The scaling constant was set at 0.84 for the width of the trial steps into the search direction of the line minimisation required by the conjugate gradient method. The geometry optimisations were considered converged when the Hellmann–Feynman forces on all atoms became smaller than 0.01 eV Å^–1^. The electronic integrations were calculated in the reciprocal space using Γ-centred Monkhorst–Pack (MP) grids^64^ of 7 × 7 × 4 *k*-points. The magnetic moments and effective Bader atomic charges were integrated within the atomic basins obtained using an enhanced grid of charge density values without lattice bias.^65−67^ We confirmed that, within the methodology used in this study, the total electronic energy was converged to within 1 meV atom^–1^.

We have employed the Visualization for Electronic and Structural Analysis (VESTA) program^68^ to generate the structural representations and we have used OriginPro^69^ to plot the distribution of probabilities and density of states.

## 3 Results and Discussion

### 3.1 Configurational Spectrum

**Figure 2:**
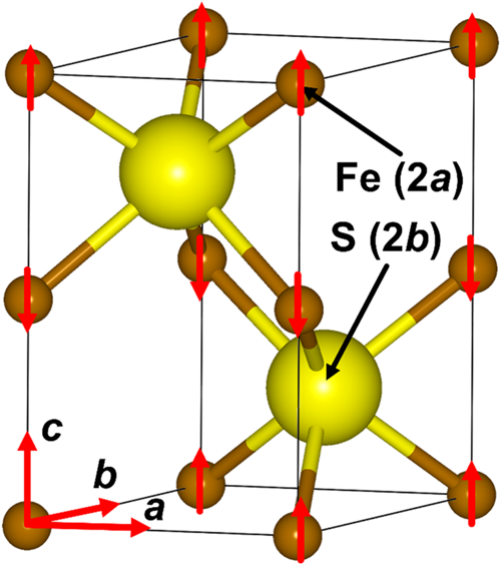
Antiferromagnetic ordering of Fe ions in the hexagonal primitive unit cell of FeS with the nickeline-type structure. Fe atoms are in brown and S atoms are in yellow.

We have first investigated the ordering of vacancies in the 25% Fe-deficient FeS with the nickeline-type structure. Our starting point was the hexagonal structure with space group *P*6_3_*mc* identified by Freund and collaborators.^24^ We have used the antiparallel magnetic ordering reported for magnetite^70^ and its isostructural sulfide counterpart greigite^60,71−74^ as well as the NiAs-like structured pyrrhotite,^18,29^ by orienting the magnetic moments of the Fe ions in a high-spin state in the same direction within each plane with the normal perpendicular to the *c*-axis and in opposite directions to the Fe ions within the adjacent planes, see Figure 2.

The study of site-disordered materials using electronic-structure techniques is challenged by the large number of possible configurations that is associated with a given supercell size. We have employed the site occupancy disorder (SOD) program,^75^ which generates the full configurational spectrum for each concentration of Fe vacancies of the supercell, and then selects the subspace of symmetrically equivalent configurations. The criterion for the equivalence of two configurations is the existence of an isometric transformation that converts one configuration into the other and the transformations considered are simply the symmetry operators of the parent structure (the structure from which all configurations are derived *via* site substitution). This method typically reduces the size of the configurational space by one or two orders of magnitude, making the problem more tractable.

**Figure 3:**
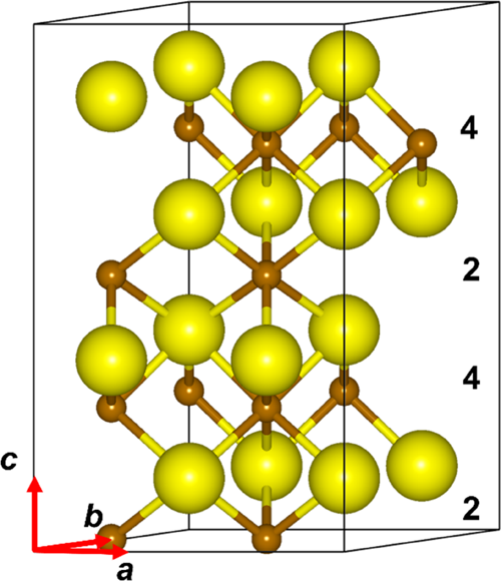
2 × 2 × 2 supercell of FeS with the nickeline-type structure and 25% Fe vacancies. Fe atoms are in brown and S atoms are in yellow.

Table 1 displays the number of primitive unit cells (*N*_C_), cell composition, the total number of configurations (*N*) and the number of symmetrically inequivalent configurations (*M*) for several supercell sizes of FeS with the nickeline-type structure and 25% Fe vacancies. We generated these supercell sizes by expanding an integer number of times the unit cell along the *a* or *c* axes. Note that the *a* and *b* axes are equivalent since this material has hexagonal symmetry, which reduces the number of possible inequivalent supercells. The primitive unit cell 1 × 1 × 1, which is the smallest possible supercell and contains a single primitive unit cell, does not have a suitable size for modelling 25% Fe vacancies, as this would entail removing half of one cation. The supercell sizes that can be simulated need to have a number of Fe cations equal to four or a multiple of four. We therefore doubled the cell in the *c* and *a* axes to obtain the smallest systems that we can model, *i.e.* the 1 × 1 × 2 and 2 × 1 × 1 supercells, respectively, which are composed of two primitive unit cells. The total number of combinations of one Fe vacancy on the four cation sites of these supercells is 4!/(3! × 1!) = 4. However, only one of these configurations is symmetrically inequivalent, as determined by the SOD code, which makes this cell size inappropriate for modelling site occupancy disorder. We then doubled the *b* and *c* as well as the *a* and *b* axes to generate supercells composed of four primitive unit cells. Despite having a total number of configurations *N* = 28, the different symmetries of the 1 × 2 × 2 and 2 × 2 × 1 supercells lead to five and three inequivalent configurations, respectively, which still is a small configurational spectrum for the purposes of our study. It is worth noting that as the 1 × 2 × 2 supercell breaks the symmetry of the parent cell, its five inequivalent configurations are not strictly able to describe the hexagonal FeS material with 25% Fe vacancies. Finally, we doubled the three Cartesian axes of the primitive unit cell to obtain the 2 × 2 × 2 supercell, which ensured maintaining the same symmetry of the parent cell. This supercell size, which contains eight primitive unit cells and is characterised by 1820 total configurations, has 38 inequivalent combinations, making it appropriate for simulating temperature-related observable averages. Figure 3 represents the structure of one of the configurations of the 2 × 2 × 2 supercell containing full Fe layers alternating with layers with 50% Fe vacancies. We did not generate the configurational spectrum of larger supercells, such as 1 × 1 × 3 or 1 × 1 × 4 or any of their alternatives, as the former would lead to cells with a number of Fe cations different to a multiple of four and the later would lead to cells with a prohibitively large total number of configurations in the complete configurational space for 25% Fe vacancies.

**Table 1:** Number of Primitive Unit Cells (*N*_C_), Total Number of Configurations (*N*) and Number of Symmetrically Inequivalent Configurations (*M*) for Several Supercell Sizes of FeS with the Nickeline-Type Structure and 25% Fe Vacancies

supercell size	*N*_C_	cell composition	*N*	*M*
1 × 1 × 1	1	Fe_1.5_S_2_		
1 × 1 × 2	2	Fe_3_S_4_	4	1
2 × 1 × 1	2	Fe_3_S_4_	4	1
1 × 2 × 2	4	Fe_6_S_8_	28	5
2 × 2 × 1	4	Fe_6_S_8_	28	3
2 × 2 × 2	8	Fe_12_S_16_	1820	38

Table 2 lists the number of Fe atoms in each of the four layers for each configuration of the 2 × 2 × 2 supercell of FeS with the nickeline-type structure and 25% Fe vacancies. We also report the degeneracy (Ω) for each configuration, which measures how many times they are repeated within the complete configurational space, their space groups and energies (*E*_m_) relative to the lowest energy configuration. Table 2 represents a multiconfigurational model of vacancy ordering in nickeline-structured Fe_0.75_S, which is able to explain two extreme scenarios, *i.e.* the fully ordered and disordered systems. The former scenario is observed if one of the configurations is much more thermodynamically stable than the rest of the configurational spectrum. The latter case occurs if the energy range of the configurational spectrum is very small or if they are comparable to the thermal energy at the equilibrium temperature. Intermediate degrees of disorder can also be predicted depending on the equilibrium temperature and the scattering of configurational energies. We found that 29 inequivalent configurations have the least symmetric space group *P*1, with degeneracies ranging from Ω = 12 to 96. The monoclinic space groups *Cm* and *Cc* are observed in six and two configurations in the reduced space with degeneracies between Ω = 4 and 48 for the former and Ω = 48 for the latter. There is only one single inequivalent configuration with the orthorhombic space group *Cmc*2_1_ that repeats four times in the complete configurational space. The configurations with the space groups *P*1 and *Cm* can be described by structures containing different combinations of none, one, two, three or four Fe vacancies within the cation layer. The structures with the space group *Cc* have two Fe vacancies in every other cation layer (2 4 2 4) and, alongside the structure with the space group *Cmc*2_1_, one Fe vacancy in each cation layer (3 3 3 3). Note that the notation with four digits within parentheses is used to represent the number of Fe ions in each layer of our 2 × 2 × 2 supercell. The first, second and third most stable configurations have the space group *P*1 with their energies differing by 0.02 and 0.09 eV f.u.^–1^, respectively, which suggests that some degree of disorder is very likely at room temperature. The ground state configuration displays the most homogeneous distribution of Fe vacancies, with a single defect in each cation layer. The least stable configuration, which has the space group *Cm* and is 0.80 eV f.u.^–1^ less thermodynamically stable than the ground state structure, displays the maximum segregation of Fe vacancies, with all of them concentrated within a single cation layer.

**Table 2:** Number of Fe Atoms Per Layer, Degeneracy (Ω), Space Group and Energies (*E*_m_), with Respect to the Lowest Energy Configuration, for the Fully Ordered Configurations in the 2 × 2 × 2 Supercell of FeS with the Nickeline-Type Structure and 25% Fe Vacancies

Fe atoms layers^–1^				Ω	space group	*E*_m_ (eV f.u.^–1^)
3	3	3	3	24	*P*1	0.00
2	4	2	4	12	*P*1	0.02
2	3	4	3	48	*P*1	0.09
1	4	3	4	16	*Cm*	0.11
2	2	4	4	24	*P*1	0.13
2	3	3	4	48	*P*1	0.14
2	4	3	3	48	*P*1	0.14
3	3	3	3	96	*P*1	0.14
2	4	2	4	48	*Cc*	0.14
3	3	3	3	24	*P*1	0.16
2	3	3	4	48	*P*1	0.17
2	4	3	3	48	*P*1	0.17
3	3	3	3	48	*Cc*	0.18
2	3	3	4	96	*P*1	0.26
2	3	3	4	96	*P*1	0.26
2	4	3	3	96	*P*1	0.29
2	3	4	3	96	*P*1	0.32
2	3	4	3	96	*P*1	0.32
3	2	3	4	48	*Cm*	0.32
3	3	3	3	48	*P*1	0.33
3	3	3	3	4	*Cmc*2_1_	0.35
2	3	4	3	48	*P*1	0.36
2	4	3	3	96	*P*1	0.36
1	4	3	4	48	*Cm*	0.37
1	3	4	4	16	*P*1	0.38
2	3	3	4	48	*P*1	0.40
2	3	3	4	48	*P*1	0.42
2	4	3	3	48	*P*1	0.42
3	3	3	3	12	*P*1	0.42
1	4	4	3	16	*P*1	0.42
2	4	3	3	48	*P*1	0.45
2	2	4	4	96	*P*1	0.48
2	4	2	4	12	*P*1	0.49
3	2	3	4	48	*P*1	0.56
1	4	4	3	48	*Cm*	0.59
1	3	4	4	48	*Cm*	0.62
2	2	4	4	24	*P*1	0.78
0	4	4	4	4	*Cm*	0.80

**Figure 4:**
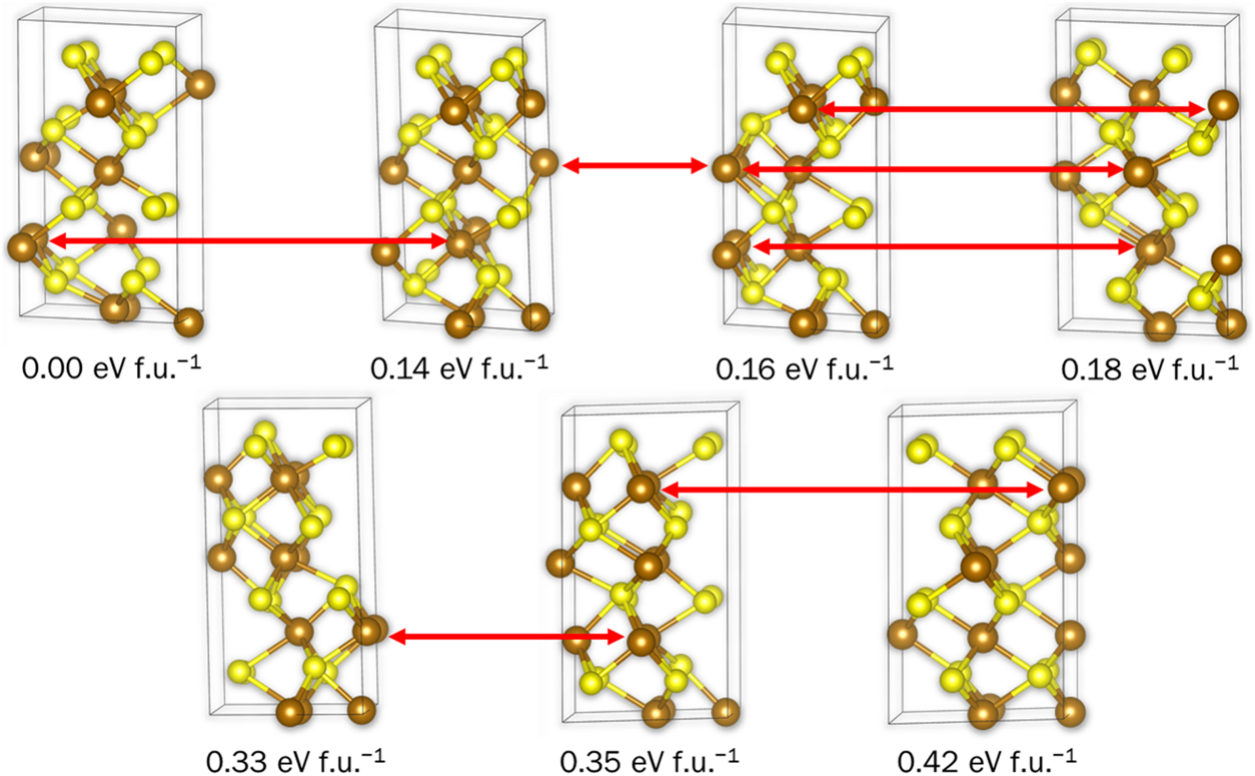
Symmetrically inequivalent configurations with one Fe vacancy per layer in the 2 × 2 × 2 supercell of FeS with the nickeline-type structure and 25% Fe vacancies.

Our analysis indicates that the distribution of a specific number of Fe vacancies over the cation layers is not exclusive to a particular space group. For example, Figure 4 depicts the seven inequivalent configurations with a single Fe vacancy in each cation layer (3 3 3 3). Five of the (3 3 3 3) configurations have the space group *P*1, with the other two having the space groups *Cc* and *Cmc*2_1_. We can clearly see that in the ground state configuration and the configuration at +0.16 eV f.u.^–1^, the vacancies are distributed in pairs, with defects in two consecutive Fe layers sharing the same *x* and *y* coordinates. Three Fe vacancies share the same the *x* and *y* coordinates in the configurations at +0.14, +0.18 and +0.33 eV f.u.^–1^ above the ground state energy. All the defects are located at the same *x* and *y* coordinates in the configuration with 0.35 eV f.u.^–1^, whereas they share the same *x* and *y* coordinates in alternating layers in the least stable (3 3 3 3) configuration.

To provide further insight into the better thermodynamic stability of the ground state (*P*1–3 3 3 3) configuration, we discuss trends in the S–Fe bond distances, S–Fe–S bond angles and magnetisation of saturation. We found that different distributions of Fe vacancies significantly affect the local geometry of Fe and S atoms in the iron sulfide with the NiAs-like structure. While all the Fe atoms remain coordinated in distorted octahedra, the bond distance becomes different for each of the neighbouring S atom, which move slightly owing to the formation of the cation vacancies. The bond distance in the stoichiometric FeS with the NiAs-like structure is 2.44 Å before geometry optimisation, whereas the average bond distance decreases to 2.26 Å, with the shortest value of 2.16 Å and the longest of 2.41 Å observed in the ground state (*P*1–3 3 3 3) configuration. Despite the average bond angle for S–Fe–S remaining very close to 90°, we found that in the stoichiometric material they differ by less than 1° for each set, since the *z* coordinate of the S atom has been displaced by 0.01 from the fractional position of 0.25 in the perfect NiAs structure. However, the formation of the Fe vacancies noticeably increases the distortion of the octahedra with the S–Fe–S angle of the different sets observed to lie between 82.33 and 99.26° in the ground state (*P*1–3 3 3 3) configuration. We found that the seven (3 3 3 3) configurations are antiferromagnetic, and therefore do not display magnetisation of saturation. However, the (*Cm*–0 4 4 4) configuration showing full segregation of the Fe vacancies, which is ferrimagnetic, has the largest magnetisation of saturation of 0.55 μ_B_ f.u.^–1^ as one of the Fe layers is missing. The magnetisation of saturation of the least thermodynamically stable (*Cm*–0 4 4 4) configuration suggests that Fe is in the low-spin configuration, which is discussed in Section 3.4.

### 3.2 Thermodynamics of Ordering from Canonical Statistical Mechanics

In this section, we discuss the vacancy ordering in terms of the probability (*P*_*m*_) of occurrence of each inequivalent configuration *m* with 25% Fe vacancies as a function of the equilibration temperature *T*. We have used Boltzmann statistical mechanics to estimate the probability from the subspace of the symmetrically inequivalent configurations as
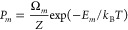
1where *k*_B_ is the Boltzmann constant. *Z* is the configurational partition function, which is obtained from
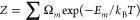
2and normalises the sum of probabilities.

**Figure 5:**
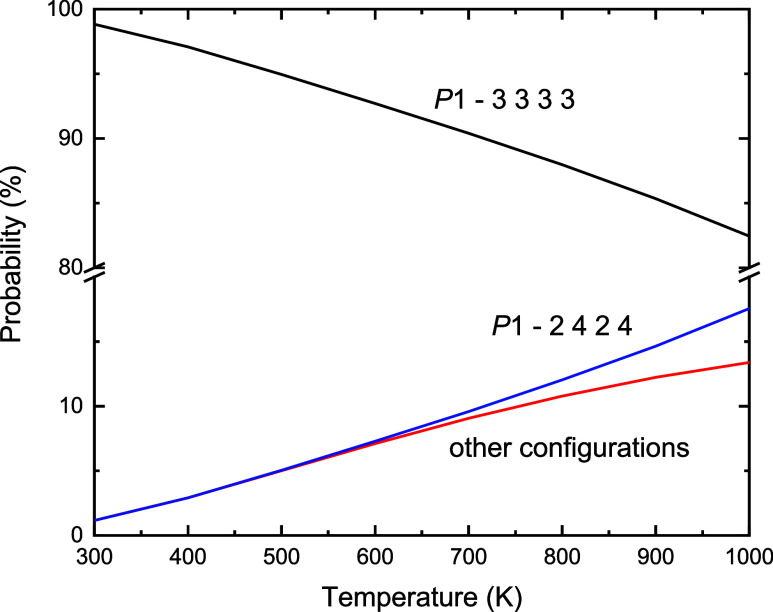
Probability of the configurations as a function of temperature for the 2 × 2 × 2 supercell of FeS with the nickeline-type structure and 25% Fe vacancies.

Figure 5 displays the probabilities of the ground state configuration (*P*1–3 3 3 3), the second most stable configuration (*P*1–2 4 2 4) and the sum of the rest of the configurations as a function of temperature. Our model suggests that the (*P*1–3 3 3 3) configuration has the largest probability of 98% at 300 K, which experiences a slow exponential decay with a negative curvature to 82% at 1000 K. The probability of occurrence of the (*P*1–2 4 2 4) configuration, which remains as the second most stable option throughout the range of temperatures considered, suffers a slow exponential growth with positive curvature from 1% at 300 K to 17% at 1000 K. The cumulative probability of the rest of the configurations also undergoes an exponential growth, but slower than for the (*P*1–2 4 2 4) configuration and with a negative curvature, resulting in 12% at 1000 K. At 625 K, which is the typical temperature of the deposition of the 25% Fe-deficient FeS thin film on the Au(111) surface,^24^ the ground state configuration has a 90% of probability, whereas the (*P*1–2 4 2 4) structure and the rest of the configurations account for approximately 5% each. This suggests that the 25% of Fe vacancies have 90% of ordering at the preparation temperature, with the 10% of disorder requiring an ensemble of the other configurations for its description. Experimental evidence shows that a temperature of 625 K is needed to promote well-ordered growth of the thin film, whereas temperatures above 750 K facilitated dewetting and decomposition, and we therefore do not consider higher temperatures in our discussion. Our simulations imply that hexagonal FeS with the nickeline-type structure and 25% Fe vacancies displays 90% ordering with the defects homogeneously distributed over the cation layers. The other 10% of the distribution of probabilities is made of all configurations but one with the Fe vacancies located in every cation site, in agreement with the available data.^24^

At this point, it is worth noting that XRD measurements are not usually employed for the structural characterisation of thin films. However, XRD can be used with confidence if the *R*-value, which measures the quality of a fit during the structural refinement, is small enough and comparable to that observed in single-crystal samples.^76^ In the particular case of FeS with the nickeline-type lattice and 25% Fe vacancies, the reported static disorder is 0.15 Å,^24^ which provides an indication of the uncertainty in the positions of Fe and S within the film. The modest static disorder reported experimentally can denote small and systematic deviations from the special positions imposed by the chosen space group. Several defective FeS polytypes with the nickeline-type lattice are able to form superstructures, where only the *a* and *b* axes are doubled while *c* remains identical. The disorder of the Fe vacancies of such superstructures can induce small deviations of the cations from their ideal positions that can be explained by the 10% disorder found in our study.

### 3.3 Average of the Observable Properties

In this section we discuss the structural and magnetic properties of FeS with the nickeline-type structure and 25% Fe vacancies. The prediction of a commensurable, albeit small, disorder in our material allow us to simulate observable properties as the average of the values calculated for their corresponding symmetrically inequivalent configurations.

Any property *A*_*m*_ defined for each configuration *m* can be averaged over the configurational ensemble as
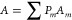
3but the interpretation of this average should be done carefully. When the averaging of system properties is performed in the reduced space of inequivalent configurations, each configuration *m* represents a set of Ω_*m*_ equivalent configurations, and therefore the property *A*_*m*_ must be the same for all configurations in that set. For example, if *a*_*m*_ and *b*_*m*_ are the equilibrium cell vectors for each inequivalent configuration *m*, the average value of the cell parameter *a* corresponding to the disordered crystal cannot be calculated as the direct average of the |*a*_*m*_| values, as this result could be different from the direct average of the |*b*_*m*_| values, breaking the rotational symmetry of the hexagonal cell. Therefore, we have calculated the *a* cell parameter as^75^
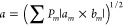
4since the absolute value of the vector product *a*_*m*_ × *b*_m_ is invariant within a set of equivalent configurations. The *c* cell parameter was obtained directly by configurational averaging of the |*c*_*m*_| values.

Table 3 shows our averaged *a* and *c* parameters for each composition as a function of temperature. The average *a* parameter decreases with temperature, whereas the average *c* lattice edge increases. Our calculations predict average lattice parameters approximately 0.2 and 0.1 Å smaller than the experimental *a* and *c* values, respectively, reported for FeS with the nickeline-type structure and 25% Fe vacancies. The slow decay of the lattice parameter with temperature can be explained based on the Fe-vacancy distribution over the cation layers in the inequivalent configurations and their probability distribution as a function of temperature. The ground state configuration (*P*1–3 3 3 3) has the largest degree of homogeneity in the distribution of the Fe vacancies, whereas the least energetically stable configuration (*Cm*–0 4 4 4) displays complete segregation of the Fe vacancies, with the remaining configurations showing intermediate degrees of distribution of the defects. In the extreme cases, where the cell has all the Fe vacancies distributed over the same layer, the *c* parameter is smaller than in the cell with a homogeneous distribution of the defects, as this effectively eliminates a cation layer from the material. Moreover, in the ground state configuration (*P*1–3 3 3 3) with a homogeneous distribution of the Fe vacancies, each S layer has six dangling bonds, with the under-coordinated soft anions becoming deformed and expanding in the crystallographic *ab* plane. The least stable configuration (*Cm*–0 4 4 4), which has no dangling bonds in 50% of the S layers, has a smaller *a* parameter than the (*P*1–3 3 3 3) configuration as the fully coordinated soft anions become more spherical. The under-coordinated S atoms that are segregated in two consecutive anion layers become stabilised by London dispersion forces and thus do not expand in the *ab* plane. As mentioned earlier, the initial magnetic configuration was antiferromagnetic, where the magnetic moments of the Fe cations were set parallel within each cation layer and with opposite spin direction in consecutive layers. The atomic magnetisations were allowed to relax during geometry optimisations, which reached the value of 0 μ_B_ f.u.^–1^ for the ground state (*P*1–3 3 3 3) configuration, since they cancel out between two consecutive cation layers. Our results suggest that temperature has a negligible effect on the magnetic moments of FeS with the nickeline-type structure and 25% Fe vacancies.

**Table 3:** Average of Observables, *i.e.*, Lattice Parameters (*a*), and (*c*), and Magnetic Moments (*M*_S_) for Selected Temperatures in the 2 × 2 × 2 Supercell of FeS with the Nickeline-Type Structure and 25% Fe Vacancies

*T* (K)	*a* (Å)	*c* (Å)	*M*_S_ (μ_B_ f.u.^–1^)
100	3.283	5.669	0.000
200	3.283	5.669	0.000
300	3.283	5.670	0.001
400	3.282	5.672	0.002
500	3.281	5.675	0.003
600	3.280	5.677	0.004
700	3.278	5.679	0.004
800	3.277	5.681	0.004
900	3.276	5.683	0.003
1000	3.275	5.685	0.001
exp^24^	3.477	5.790	

### 3.4 Density of States

**Figure 6:**
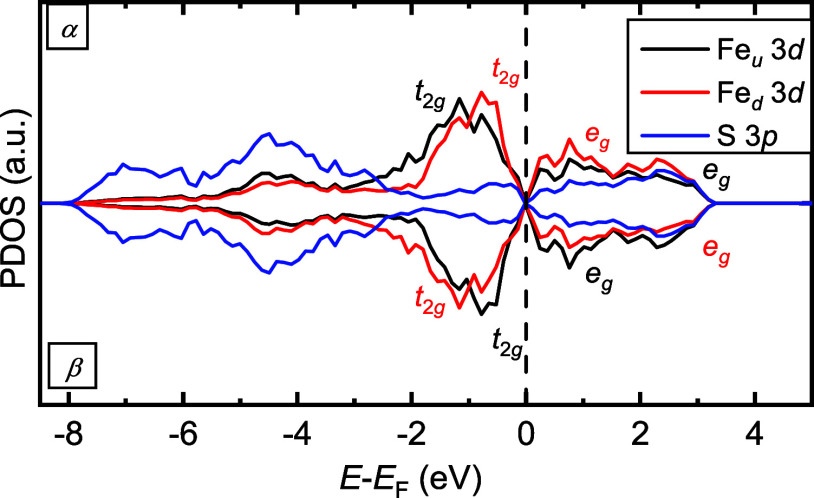
Atomic projections of the spin-decomposed density of states (PDOS) for the ground-state (*P*1–3 3 3 3) configuration of Fe_0.75_S with the NiAs-like structure. *α* and *β* denote the majority and minority channels of the spins, respectively. Fe_*u*_ and Fe_*d*_ stand for the iron atoms in adjacent planes with their magnetic moments oriented in opposite directions.

The density of states (DOS) shown in Figure 6 indicates that the ground-state (*P*1–3 3 3 3) configuration of Fe_0.75_S with the NiAs-like structure is semimetallic, as there is a small density of states at the Fermi level (*E*_F_) and no band gap. In the majority spin channel (*α*) of Fe_*u*_ cations (*t*_2*g*_), within an arbitrary cation layer with the normal perpendicular to the *c*-axis, the valence bands are dominant at −1.2 eV. In the minority spin channel (*β*), these bands shift to −0.8 eV. Both spin channels of the unoccupied *e_g_* bands for Fe_*u*_ ions are located between *E*_F_ and 3.2 eV. We observe a similar trend in the projected density of states (PDOS) of the Fe_*d*_ cations in layers adjacent to Fe_*u*_. The main difference between the PDOS of Fe_*u*_ and Fe_*d*_ lies in the opposite orientation of their magnetic moments. The slight asymmetry in the PDOS of the cations is consistent with a low-spin electron configuration of Fe^3+^: *t*_2*g*↑_^3^*t*_2*g*↓_^2^ and Fe^2+^: *t*_2*g*↑_^3^*t*_2*g*↓_^3^, which combined contributions are plotted in Figure 6. To ensure that Fe_0.75_S with the NiAs-like structure remains charge-neutral, we have assumed that Fe^3+^ and Fe^2+^ have a 2:1 ratio. The perfectly symmetric PDOS of S are spread between −8.0 and −2.5 eV below the Fermi energy, indicating a filled *p* shell. Additionally, there is a minor contribution from the S *p* orbitals in the conduction band, which appears strongly hybridised with the Fe *e_g_* states between 1.5 and 3.2 eV. Despite the similar Fe^3+^ to Fe^2+^ ratio, the Fe cations in Fe_0.75_S with the NiAs-like structure adopt a low-spin state. In contrast, its quasi-polymorph greigite (Fe_3_S_4_) exhibits a high-spin electron configuration, as evidenced by the pronounced asymmetry in its PDOS.^74^

## 4 Conclusions

We have performed a theoretical investigation into the stability and structural behaviour of FeS with the nickeline-type lattice and 25% Fe vacancies. We found that the 2 × 2 × 2 supercell of the primitive hexagonal cell with Fe deficiency contains a large enough number of symmetrically inequivalent configurations to effectively simulate the structures, energies and averages of the observable properties. The ground state configuration displays a homogeneous distribution of Fe vacancies, whereas the least stable configuration comprises full segregation of the vacancies. Canonical statistical mechanics indicate that under the synthesis conditions, the material is disordered to approximately 10%, which is compatible with experiments. As the temperature increases, the calculations also show a decrease in the cell parameters of the Fe-deficient material, which are slightly underestimated with respect to experiments. Our simulations also confirm that the Fe-deficient material is essentially antiferromagnetic at any temperature. The most stable configuration of the Fe-deficient iron sulfide is described as a semimetallic. Our work provides important atomic-level insight into the origin of the larger order of 25% Fe vacancies in FeS with the nickeline-type structure, which has implications in the design of novel catalysts for CO_2_ conversion. Future work will focus on the simulation of different surface terminations, reconstructions, Fe vacancy concentrations and the chemical reactivity.

## Data Availability

All data created during this research are provided in full in the results section of this paper.
